# Processing insect abundance: trading and fishing of *zazamushi* in Central Japan (Nagano Prefecture, Honshū Island)

**DOI:** 10.1186/s13002-015-0066-7

**Published:** 2015-11-11

**Authors:** Nicolas Césard, Seiji Komatsu, Akihisa Iwata

**Affiliations:** Graduate School of Asian and African Area Studies (ASAFAS), Kyoto University, 46 Shimoadachi-cho, Yoshida Sakyo-ku, Kyoto, 606-8501 Japan; CNRS/MNHN Laboratoire d’Eco-anthropologie et Ethnobiologie, UMR 7206 - HNS Muséum national d’Histoire naturelle - Site du Musée de l’Homme, 17 place du Trocadéro 75116, Paris, France

**Keywords:** Entomophagy, Wild harvesting, Technique, Ethnoentomology, Japan

## Abstract

**Background:**

This article presents the links between technique, commerce and consumption in fishing for zazamushi, a mixture of aquatic insect larvae sold as food souvenirs in Japan. Since zazamushi are mainly collected for economic reasons, we suggest that demand for them has incited technical development among collectors in order to fish more insects.

**Methods:**

Several fishermen and traders were interviewed in semi-directed interviews about their practices and knowledge. To understand the passage from a faunal composition to a commercial composition, our research follows a fishing session closely, as well as the selection of insects that follows it. The insects collected were separated from inanimate matter, then identified, counted and weighed at each stage of the process.

**Results:**

Our results suggest that the current technique corresponds to an evolution in subsistence and recreational collecting towards a more systematic fishing of the insects, the aim of which is commercial. In their response to trade issues, the collectors have moved away from the banks to fish the insects in the river current, thus increasing the amount of one species captured compared to another. Although the technique is efficient (and similar to other harvesting techniques), it requires the thorough sorting of organic debris and insects (in our example, the catch contains approximately 78 % of inanimate matter and 22 % of insects, of which 3.29 % are retained for consumption, i.e., less than 2 out of 100 insects). The selection of insects to be consumed takes place mainly during cleaning. This stage depends on traders and reflects the different compositions sold as souvenirs.

**Conclusions:**

Our research shows that the consumption of insects is not explained just by ecological factors that are favourable or unfavourable, but also by technological and economic factors related to their commerce. It suggests that the traders have gradually established the insects that are currently sold as zazamushi and that this commercial development may have had an influence on the preference for insects consumed. It also shows that the cleaning of the insects constitutes an important stage prior to their consumption, one that should not be underestimated.

## Background

Around 2000 species of insects are currently known to be consumed as human food [1] and probably more are suitable for animal feed. Entomophagy arouses increasing scientific and agrifood interest, mainly in terms of food safety [2–4]. Insects form part of the solution currently being developed for feeding a growing global population. Companies that produce and process insects for animal feed and to a lesser extent for human food are being created in both temperate and tropical countries. Insects are tested and developed for production by farming. Many of these intensive production practices find their origins, even their current legitimacy, in local consumption practices that are often ancient or have been forgotten or neglected for a long time. In the same way, their production targets and the use of technologies adapted for insects are not far from the methods implemented by certain collectors around the world to understand the relationship between insects and their environment and to take advantage of them. Unlike the production of insects by farming, they rely essentially on collecting edible insects from their environment.

### Insects in large numbers

Most insects are small, have narrow distributions in relation to resource requirements, short generation times, high reproductive rates and often large population sizes [5]. The presence of the insects harvested and consumed in the greatest numbers is connected to several biotic and abiotic factors and their interactions and to physical environments that are often specific [6]. Although certain insects, of which several are non-gregarious, are gathered by humans in an opportunistic manner when they are encountered during other activities, most edible insects are harvested where they are concentrated, in particular when they appear in large numbers at certain periods. The majority of massive insect migrations, the most spectacular ones, stand out for their periodicity, their persistence in time and their extent.

However, if the abundance allows *a priori* one or several harvests, it only partly explains their occasional or regular consumption. Other factors cause a species associated with a habitat, or one that is periodically abundant, to be sought or expected for harvest and consumption. These factors are often highly variable and interdependent.

When the frequency of appearance of species is linked to the assurance of abundant harvests, it offers some *predictability* and constitutes a major motivation for preparing for the harvest. In many countries in which the insects are consumed or used for animal feed, the periods of abundance (when they are regular) and the places where they are likely to be found are generally known, and due to this, the massive appearance of insects corresponds to the main harvesting periods [7]. The predictability of many outbreaks is associated locally with seasons or certain months of the year. An abundant species can thus follow another. Just as it is common for several edible species to be present at the same time, but in different proportions (for an example of seasonality in Vientiane, Laos [8]). The consumption periods do not then always correspond to peaks in abundance (whereas the contrary is common); certain species can thus be consumed all year round in smaller quantities, or because they have been treated for storage and consumed later.

The result of a harvest, even the presence itself of a species on a site can depend, in part, on the collectors knowledge of the insects and their environment (like in the case of semi-cultivation), but also on the *skills* associated with this knowledge and the *technologies* implemented in these harvests. These harvesting skills and techniques are adapted to the specific needs of the species, its population and the physical environment of the prospected sites. Know-how are likely to vary depending on collectors, their interests and their aims. Indeed, if everyone can take advantage of the resource as soon as it is accessible, not all the collectors share the same experience and the same knowledge. In addition, access to knowledge or a technology does not guarantee its optimal use. For example, certain knowledge may prove inadequate to the aims of the harvest. In the same way, certain tools may not respond to expectations (and those tools can be perfected to collect more insects, as we will see in our example), while others can be under or badly used.

If, most of the time simple knowledge and techniques are sufficient to capture the insects in the quantities needed, there exists extensive know-how to benefit from their presence. The most efficient methods rely on knowledge about the insect and its environment, as much as to identify the sites as to find the most appropriate tools and methods of harvesting. To ensure an abundant harvest, the collectors can intervene in the environment mainly in two ways: by exploiting it at best, and also by manipulating it in anticipation of future harvests.

### Semi-cultivation and expert harvesting

One way of ensuring the presence of insects in their environment, even to anticipate their arrival where they are expected, is to foster their biological development: on the one hand, by influencing the environmental conditions and on the other, by implementing good (or better) collecting practices, some of which could lead to the sustainable management of the resource. Some local biological and ecological knowledge, traditional or indigenous, but also recent research has led to the manipulation of habitats, more rarely of the insects to result in *semi-cultivation* [9].

Another way of facilitating both accessibility and availability of the resource when it is abundant, but without the harvesters always being able to favour or develop it (due to the natural conditions or due to lack of knowledge and/or means) is to implement efficient harvesting techniques that rely on a good understanding of the ecological environment (more perhaps than the insect’s biological needs). While simple harvests, such as the collecting of one insect after another, are more closely related to gathering or picking, several collectors around the world have developed technical skills with varying degrees of complexity in order to capture a larger quantity of insects, such as fishing and hunting tools [10–12] and sophisticated traps [13].

The aim of these harvests is to capture a maximum number of insects, but also to make the harvest easier especially in view of the conditions of the site and the environment. The efficiency of the skills developed in these practices is found in the development of tools or resources adapted to the practice of regular harvests. If these methods can follow and benefit from the abundance of semi-cultivation (or breeding), they try above all to take advantage of the presence of the species for a limited amount of time: that of the abundance of insects at a given moment, but also for the collectors, that of the time spent on site.

The desire to make as much as possible from the presence of a resource is not justified only by food related, biological or technical reasons. Among the motives that explain that an abundant resource is exploited, the economic criterion is essential. If edible insects are harvested to be consumed directly, they are also collected for sale. Today, many species, especially insect outbreaks, are not collected for their nutritional qualities -or no longer only for this reason- but for the profits they contribute to their collectors and those that sell them. In many countries the abundance of insects when they are or can be consumed represents a revenue opportunity that is sometimes significant. The profit from commerce justifies the expenditure connected to the harvest, whether it is material investment or physical effort and the time spent harvesting and preparing the insects for sale.

In light of the above, this article presents the links between technique, trade and consumption in fishing for zazamushi, a mixture of aquatic insect larvae sold as food souvenirs in Japan. Since the insects are mainly collected for economic reasons, we suggest that the demand for zazamushi has caused in collectors a technical development to catch (or harvest) more insects and to respond to trade.

### The consumption of insects in Japan

The consumption of insects around the world is often presented for its protein intakes. But the insects are mostly consumed for their taste [14] or rejected because they have a bad flavour. Indeed, few people consume insects that they do not find good on a regular basis. Many insects are cooked and prepared with other ingredients (spices, sugar, oil, etc.), for their taste, but also for their specific texture. In some countries or regions where insects are or were traditionally consumed but where other food is more easily available, for purchase in particular, insects are no longer consumed on a regular basis, but periodically as so-called favourite foods. This is the case in Japan [15], but also in other countries. In Thailand, the migrations of Isaan workers from Northeast Thailand have moved the consumption of insects towards big cities like Bangkok [16]. Many insects are no longer gathered in the environment, but are now produced in farms, before being packaged for sale and consumption in the form of snacks [17].

Japan is one of the few temperate countries where insects were, or are still, consumed. At the beginning of the 20th century, Miyake [18] recorded 55 insects recognized as food and 123 insects for medicinal uses in the entire archipelago. More recently Mitsuhashi [19] listed at least 117 insect species traditionally used as food (in 45 of 47 prefectures in 1986 according to Nonaka [20]). In the furthest inland regions, insects varied the diet and were important sources of protein [15, 19]. They were collected and consumed during the harvesting season but also simmered with soy sauce and sweet rice wine (or sugar) (in *tsukudani*) to be preserved for longer. Today the insects are mainly consumed as “nostalgia” [15] or as “homeland food” (*kyoudo-shoku*) by locals and domestic tourists. Packaged in cans, in glass bottles or displayed in punnets the insects are sold year-round as souvenirs (*omiyage*) in boutiques that specialize in regional products (*meibutsu*). They are appreciated by older people, but they are above all consumed by the family during the year end festivities and given as gifts to relatives or friends who have come from the city. In some Japanese prefectures, insects like grasshoppers or silkworms can also be found as snacks in ordinary supermarkets and sometimes restaurants.

In the city of Ina in the south of the Nagano prefecture, at the centre of Honshū Island, four types of insects are thus sold in boutiques: *inago* (a variety of grasshoppers, *Oxya yezoensis*, sometimes *Oxya japonica*), *hachinoko* (traditionally, the larvae and pupae of social wasps, *Vespula* spp.), *kaiko* (the nymphs of the domestic silkworm, *Bombyx mori*) and *zazamushi* (a mixture of various aquatic larvae but today mainly *Stenopsyche marmorata*). With the exception of silkworms, the insects sold are collected in the wild (like the grasshoppers of the paddy fields [21]). Despite a decline in the diversity of edible insect consumption and valuation with recent generations, grasshoppers and wasp larvae remain in high demand in rural Japan [22]. However, since most food insects are less abundant than in the past, and Japanese collectors are too few, current demand requires varied contributions for the traders, with insects often being sourced from outside the region in which they are consumed. Indeed, many insects sold in Ina’s boutiques, except for zazamushi, come from abroad, in particular from mainland China, before being mixed in more or less variable proportions with Japanese insects.

The article here looks at fishing for zazamushi in the Tenryū River, as an expert harvest, and at its trade which, as we will show, is inseparable. The generic term of “zaza-mushi” refers to the onomatopoeia “zaza” from “the noise of the water that runs over the stones in a shallow river that is quite fast running” [23] and the laden category of “mushi” which includes insects and depending on the context, other small animals [24]. “Zazamushi” refers first to all the larvae of small freshwater benthic animals that live hidden under stones. The rather wide category includes mainly insects such as stoneflies (Order: Plecoptera), caddisflies (Order: Trichoptera), dobsonflies (Order: Megaloptera), dragonflies and damselflies (Order: Odonata) but also small crustaceans (Gammaridae, *Asellus*), fish (gobies) and several “worms” (Phyla: Annelida, Platyhelminthes). By extension, the term also refers to insects consumed and traded as food souvenirs. Several publications have shown that the fauna composition of zazamushi in the Tenryū River changes depending on the location and period of their collection ([25], Table 1), in the same way that it is likely to vary from one year to another at the same location [26]. One of the first observations of the composition of the insects in their habitat [27] suggests to certain authors [15, 23] a general decrease since the first quarter of the 20^th^ century in the number of Plecoptera parallel to an increase in the number of Trichoptera. The product destined for human consumption has also changed over time.

**Table 1:** Commercial compositions by time (number of insects %)

Class	Order	Species	1934 [34]	1988 [29]	2007 Kaneman (can) [25]	2014 Tsukahara (bottle)	2014 Green Farm (bottle)
Insects	Ephemeroptera				0.6	0.9	−
	Trichoptera				98.2^a^		
		*S. marmorata*	74.2	62.3		91.7	100
		Others		34.1		−	−
	Plecoptera		21	−	−	−	−
	Megaloptera	*P. grandis*	3.2	2.6	1.2	1.8^b^	−
	Others		1.6	−	−		−
	Coleoptera			−	−	5.5^c^	
Others			−	1.0	−	−	−

^a^Including *Stenopsyche marmorata*

^b^Two instars of stage 3

^c^Family: Psephenidae

The exceptional density of the benthic fauna of the Tenryū River originates in an ecosystem that is especially rich in nutrients borne by the inputs of the Lake Suwa upriver. Several authors indeed explain the consumption of zazamushi in the region by the abundance of insects in the river, and the changes in the composition of the insects that are sold mainly by ecological arguments. According to Murakami and Yaguchi [25], the consumption of caddisfly larvae and the transition in consumption from the 1930s follow the eutrophication of Lake Suwa and the change in the dominant species of fauna. Others also mention the building of dams and weirs and the effect of pollution in the disappearance of Plecoptera [26], the analysis of a sample in the river recorded in 1957 0.2 % individuals of *Kamimuria tibialis*, [28, 29] and more indirectly in the current interest for consuming Trichoptera [25]. They conclude in particular that “the large biomass supported by the supply of particulate organic matter from Lake Suwa has made it possible to commercialize canned zazamushi” [25].

The argument developed here is that the ecological explanation, although it is probably right, is incomplete because it does not take into account the role of trade and demand in the composition of the insects sold, and through trade, the development of the technique. If the nutrient supply from Lake Suwa justifies the large animal biomass in the Tenryū River, it does not explain trade in zazamushi as food souvenirs, nor more broadly the interest for consumption of insects. At best, it only allows us to understand the abundance of the resource (like water pollution could explain the decrease of certain species). In short, the ecological evolution of the lake can explain the benthic diversity of the river, but not the fact that the people fish (or don’t) or consume (or don’t) insects, and certain insects rather than others.

To understand the role of trade in fishing and the current composition of zazamushi, we will describe the ecological and technical knowledge associated with the fishing and will discuss their evolution. After presenting the specific environment in which the main insects consumed evolve, their ecology and briefly introducing the collecting and fishing for zazamushi currently being practiced and its trade, the research shall present the stages of fishing and the detailed results of a fishing process. These ethnoecological knowledge are implemented in three phases of the fishing. The first covers the search for and identification of the best sites. The second corresponds to the fishing and its techniques. The third deals with the selection process for the insects which in our example, goes from the fishing process to the various types of sorting to retain only edible insects. The discussion will show that the fishermen and traders in zazamushi select the insects that interest them, and that these choices appear in the manner in which the insects are fished and in the way in which they are selected before being cooked.

## Methods and materials

### Study area

**Fig. 1:**
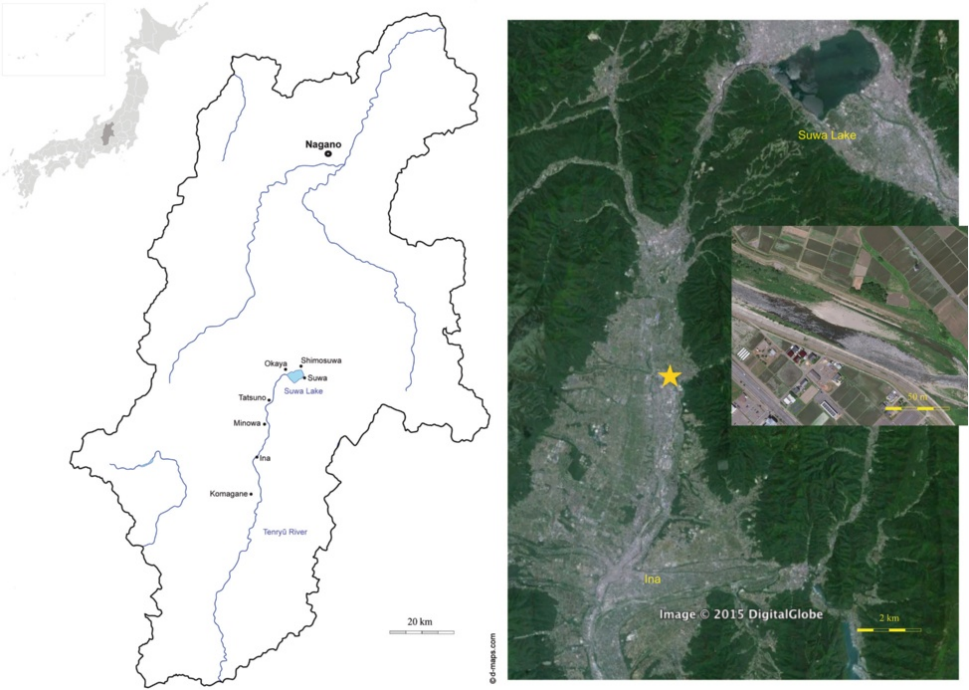
Maps of Tenryū River in Nagano prefecture with primary study site (Tables 3, 4, 5 and 7). (Adapted from Wikimedia Commons/WiJG?, d-maps.com and Google Earth 2015/Bluesky)

The study examines fishing for zazamushi along the upstream section of the Tenryū River. The context is the city of Ina (35°50’N 137°57’E) and the neighbouring localities (Nagano prefecture) (Fig. 1). With a length of 213 km, the Tenryū River begins its course at Lake Suwa close to the cities of Okaya, Shimosuwa and Suwa and runs further south into the Pacific Ocean. The region is characterized by deep valleys and by heavy precipitation (over 3000 mm per annum). The Ina valley watershed upstream is a prosperous agricultural region surrounded by the Southern Japanese Alps to the East and the Central Japanese Alps on the West.

At an altitude of 759 m, Lake Suwa is a shallow hypertrophic lake with an area of 113.3 km^2^. The lake’s average depth is 5 m (maximum of 6.8 m) but the depth of the deposits (mud layers) is significant (and favourable to the development of herbivorous chironomid larvae [30]. The area around the lake is densely populated (over 170,000 inhabitants in total). Urban and industrial activities constitute a considerable anthropogenic contribution, marked amongst other things by pollution episodes [30]. Since the end of the 1960s, microcystin blooms have been observed regularly during the summer [31] and are evidence of eutrophication of the lake [32, 33].

### Methodology

Carried out over several years, the majority of previous studies have based their analyses of the composition of the zazamushi of the Tenryū River on insects that were sold [29, 34], some comparing them with insects collected directly on site [25, 28, 29]. If comparisons with the earliest references in the literature show, amongst other things, the massive predominance of Trichoptera in the compositions sold (see in particular Nakai [29]), their results conclude with compositions that are often very different between the zazamushi consumed and those collected in the environment.

We believe that due to the different locations where zazamushi are collected (or due to the fact that locations are not specified), it remains difficult to compare these samples analysed. Indeed, although there are differences in composition between the insects fished and those sold as souvenirs, it seems to us that in order to understand these differences and to compare them, the analyses cannot rely on insects caught separately but on samples taken at the same place and at the same time.

To understand the passage from a faunal composition to a commercial composition, our research follows a fishing session closely (one individual fisherman), as well as the selection of insects that follows it (Tables 3, 4, 5 and 7). It calls on the skills of the anthropology of techniques and insect ecology to understand the fishing process as a selection process not only of sites but also of the insects to keep. We provide a description of the succession fishing actions aimed at improving production and quality.

The fieldwork was conducted during two visits lasting about ten days, before and then during the fishing season (November 2013 and January 2014). Semi-directed interviews on the fishing practices, the tools used and their knowledge of the river were held with eight fishermen (of which four fishermen were active that year, that is, they had requested their fishing permit). We also contacted three traders including the two most active ones (and attended several food processing sessions), as well as the main distributor of insects commercialized in the region. Before each interview informed consent was obtained. All participants pictured provided their approval.

Three fishing sessions at different sites were followed, of which two were followed in their entirety, in the river and then in the fishermen’s homes. A sequence (four successive catches for a total of nine minutes) of the first fishing session was the subject of systematic capturing of insects at different stages; first with the help of a seine net placed behind the four-hand net to measure which insects escaped (Table 3), then by taking samples of the rejected “trash” (inanimate matter such as little stones, leaf litter, wood pieces, detritus, but also unwanted and non-visible organisms) (Tables 4, 5 and 7). Only the results of the catch from the second fishing session were examined, in particular during the insect cleaning and sorting stage (Table 6). All the insects collected during the two fishing sessions were separated from the inanimate matter, then identified, counted and weighed. We also analysed the results from two bottles of 30 g sold in 2014 (see Table 1).

### Species composition of zazamushi

The various compositions of the insects in souvenirs show the predominance of Trichoptera (mainly *Stenopsyche marmorata*), of one Plecoptera species (*Kamimuria tibialis*) and of another of Megaloptera, *Protohermes grandis*) (see Table 1). We will present briefly the ecology of the three main insects that are sold.

The larvae and pupae of *net-spinning caddisflies* are especially common in rivers with a stable bottom of gravel [35]. Those of *Stenopsyche marmorata* live in particular in rivers in streams with relatively larger substrate particle sizes (pebbles, cobbles, and boulders) [36]. The larvae tend to build retreats in substrate pore spaces (between stones) [37]. The larvae build feeding nets attached to retreats among streambed particles by spinning silky threads to form a coarse and irregular meshed net structure [38]. The principal role of the feeding net is to efficiently trap drifting detritus and facilitate foraging activities. Like the majority of Trichoptera, *S. marmorata* go through five larva stages [39], the final stage larvae reaching a size of 40 to 45 mm [37]. Present throughout the year, their life cycle depends on the temperature of the river [40]. In the same way, their feeding habits are different depending on the instars, the species shows a change in feeding habits with a transformation from fourth to fifth instar, especially having a strong tendency towards predacious feeding for fifth instar larvae [41], a change that can be explained by preparation for hibernation [42].

The larvae of *S. marmorata* feed mainly on Suspended Particulate Organic Matter (SPOM) and other sources of food are diatoms, blue-green algae, green algae and debris [43]. Their capacity to capture organic material accumulated by the river and consequently their feeding preferences vary according to the size of the net but also according to the current velocity and the streambed characteristics [44]. As scavengers the larvae play an important role in trophic relations [45], just as they also help to control the transport of organic matter in rivers [46]. The abundance and biomass of the species can reach 2000 individuals per m^2^ and 50,000 mg per m^2^, respectively [45]. Katagami et al. [33] claim that the upriver region of the Tenryū River has the largest population in the world of *Stenopsyche marmorata*. This population apparently contains important concretions of microcystins (in particular young larvae), but the toxicity is apparently not a danger for human consumption [33]. Due to their blue-green colour, the locals call them “green insects” (*ao-mushi*).

*Kamimuria tibialis* has been identified as one of the most collected species of Plecoptera on the Tenryū River. Like most large sized stoneflies [47], the species is omnivorous and can vary its diet depending on the season [48]. The current in particular determines the spatial separation of Plecoptera [49]. The oxygen concentration influences distribution within a section of the river, and therefore the preferential habitat of a species can change from one period to another. In the central mountains of Japan, *K. tibialis* chooses sites with slow to intermediary current) all year round and can share slow currents in winter with other stoneflies such as *Oyamia lugubris* [50].

The species of the subfamily of the Corydalinae, *Protohermes grandis* (Megaloptera) (known as *magotaro* in Japanese), is widely distributed in Japan [51]. The carnivorous larvae of the dobsonfly dwell in stream riffles and feed on a variety of aquatic animals (mainly Ephemeroptera in the example given by Hayashi [52]). The study by Hayashi [52] suggests that *Protohermes grandis* have nine larval instars. He also shows that the final development stages are influenced by the availability of large prey (and here we assume larvae of *S. marmorata*). Larval development takes two or three years. Then, the final instar larvae leave the stream to pupate in soil on the banks. Shortly afterwards, the adult emerges and reproduces.

### Fishing zazamushi

Depending on the quantities desired and the equipment available, zazamushi are collected on the Tenryū River in two ways. The first method was probably the more common in the past (Post-WW2) and is still favoured today. It consists of catching the larvae with wooden chopsticks. To do this, the gatherers walk along the river banks in search of large stones that are submerged within their reach. They turn them over one by one, generally by hand, sometimes with the help of a claw, then with the chopsticks, they collect the larvae that they dislodge from their retreats. The collectors only stay in place for a few hours. Some gatherers can move forward 1 or 2 m into the river, but only in very shallow areas so that the larvae, once they are uncovered, do not fall or are not carried off by the current. The use of chopsticks does not require a fishing permit. Since the collectors are not registered, it is hard to assess how many of them exist. The information acquired from some presents them as quite numerous, but irregular in their activity. Those in activity gather insects during the weekends or their holidays. The quantities collected by each individual with chopsticks are generally low (less than 500 g). They are however sufficient for individual or family consumption.

**Fig. 2:**
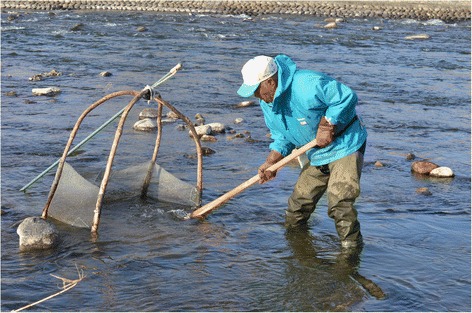
Fishing zazamushi in the Tenryū River. (Photo by N. Césard)

The second method was generally practiced in the past, but the equipment used appears to be a recent development. The collection is associated with fishing and requires a special licence from the local Fishermen’s association (*Gyogyou kyoudoukumiai*), the “licence to trample insects” (*mushifumi kyokasho*), which only people who already have a fishing licence (*saiho kyokasho*) can obtain. The method consists of catching the insects directly in the current. To do this, the fishermen use a quadrangular stainless steel net (wire mesh width 2 mm) whose sides are closed, except for one side by which the insects enter. The net is held by four rods, in wood or metal, which cross in the upper section. Derived from fishing, this type of square net, known locally as a “four-hand net” (*yotsude-ami*), is assembled by the collectors themselves and is adapted to fishing insects in the rapids. Its use in a river requires a minimum depth in order to be placed and a minimum of current to push the insects into the net (Fig. 2). As we will see, its efficiency is also its main defect: it captures the insects being sought, but also numerous other living organisms and inanimate matter.

### Marketing zazamushi

Before being sold as souvenirs, zazamushi were mainly collected and consumed by locals of the Tenryū River. The reconstitution shows two phases in the trade: a first phase when the zazamushi were collected and sold locally [34], then a second which saw the arrival of packaged insects. The sale of zazamushi as food souvenirs developed starting in 1956 when the Ina-based company Kaneman (closed in 2011) offered preserved zazamushi (for a price of JP¥55 for 100 g then) [15]. Sales seem to have been quite successful because several boutiques in the town offered them (three still today) and a fishing licence was implemented from 1960 to identify the collectors [15]. The gathering of insects became a seasonal and regular activity for some fishermen, and for those most invested, a lucrative activity (Table 2). Today, still, the fishermen can reimburse the cost of the licence (JP¥15,000/US$120) in three days.

**Table 2:** Annual number of licenced zazamushi fishermen and average catch per fisherman (source: Ina Fishermen’s Association)

Year	No. of fishermen	Total catch (kg)	Average catch per fisherman (kg)	Year	No. of fishermen	Total catch (kg)	Average catch per fisherman (kg)
1960	22	N/A	−	1992	46	1800	39.13
1961	8	N/A	−	1993	52	3100	59.61
1962–71	N/A	N/A	−	1994	78	2200	28.20
1972	14	N/A	−	1995	70	2200	31.43
1973	21	N/A	−	1996	73	2000	27.39
1974	17	N/A	−	1997	72	1200	16.66
1975	22	N/A	−	1998	63	600	9.52
1976	N/A	N/A	−	1999	33	100	3.03
1977	41	N/A	−	2000	39	700	17.9
1978	48	N/A	−	2001	41	1300	31.70
1979	40	N/A	−	2002	57	1600	28.07
1980	23	N/A	−	2003	56	1200	21.42
1981	8	N/A	−	2004	16	200	12.5
1982	40	N/A	−	2005	50	1600	32
1983	19	N/A	−	2006	27	432	16
1984	43	12,000	27.91	2007	18	N/A	−
1985	19	890	18.16	2008	42	N/A	−
1986	43	12,000	27.91	2009	28	N/A	−
1987	71	16,000	22.53	2010	30	N/A	−
1988	53	600	11.32	2011	18	N/A	−
1989	29	100	3.44	2012	24	N/A	−
1990	57	230	4.03	2013	11	N/A	−
1991	21	100	4.76	2014	13	N/A	−

Unlike other insects, zazamushi are abundant and still collected locally. However, although souvenirs and local consumption cover demand, for several years the number of fishermen invested in fishing has been falling, having reached the age limit and in the absence of new fishermen to replace them. To this is added the influence of typhoons and constructions that changes the river’s morphology, thus making the results of the catches uncertain from one year to another (Table 2).

## Results

Insect fishing for consumption focuses on the almost exclusive search for Trichoptera (caddisflies). Since 1960, the fishing season has been fixed at 3 months by the local fishermen’s association, from the beginning of December to the end of February. According to the fishermen, this period corresponds to the most suitable weeks for capturing insects for consumption because the larvae are considered to be fatter and tastier (it corresponds to the insects’ hibernation period). For many, however, the beginning of December appears slightly premature because caddisflies still retain sand in their intestines and their flavour, even after cooking, is affected by this. Most fishermen prefer to catch the insects later in December, often starting from the second week, when the weather gets very cold. The best season then extends until the end of January, even early February. In the past, collecting continued until March, even April [23]. This is sometimes still the case today for those who use chopsticks.

During winter months, the cold makes the conditions for fishing especially difficult. The fishermen stay in the current for many hours, often with water up to their knees. The bulky equipment and riverbed morphology make it difficult to move. In each sector being fished, the fishermen repeat the same gestures, before moving 1 or 2 m and starting again. Depending on the weather conditions, but also their age and motivation, the fishermen work from 3 to 5 h a day, generally in the same sector, and often 2 to 4 days a week during the fishing season. Those that sell their catch, start the day early and are more regular than occasional collectors. Most of the fishermen who were questioned begin in the late morning and finish during the afternoon, around 15.00 or 16.00, even earlier for the less assiduous. The fishermen avoid days when it rains, snows or is windy. Sunny days make fishing more pleasant, but as we will see, also facilitate the sorting of insects by “warming up” the larvae and making them more mobile.

### Searching for fishing sites

The first stage is to identify the sites to prospect. Most fishermen come from the region. They go to the river most of the year to fish, but also to ramble. Some live nearby, others pass it every day. All year round, the fishermen observe the river and its transformations, often in view of future activities. When winter comes, one month, even two before insect fishing opens, the collectors begin to search the sites to prospect seriously, those likely to have the largest number of insects. For this, they drive along the river and, from their car, identify the locations.

The first indicators are visual. From their vehicle, the fishermen observe the rapids first of all, estimating the size of the stones and strength of the current. From experience, they know that currents which are too fast have less insects and they make the four-hand net hard to handle. They therefore find slower stream riffles (*zaza*), those slightly apart from the main channel and in particular sectors of the river that are relatively flat and shallow (called *touasa*). In these sections that go from the bank to the channel, they assess the places where the stones are too small and also those where the larger rocks are too dark or smooth because they seem not to have any algae, so as to avoid them later.

From one season to another, the fishermen pay particular attention to any construction work which, to control the flow of the Tenryū River often changes the most interesting areas for insect fishing, when it doesn’t create turbidity downstream, often for several weeks (and can last up to 3 months according to the fishermen) which provokes siltation of the sites that are favourable for insects. Generally, in a river where embankment works are common, most fishermen favour the most natural sections, those where the river has returned to a normal course leaving on its way between the banks, low water levels and high water levels.

Accessibility is also a major factor in choosing sites: the fishermen must be able to park near the site, but also be able to get down easily from the bank to the river, often contained by concrete dikes several meters high. Most fishermen catch the insects in the sections of the river they know best, rarely far from their homes. In good years, when the optimal conditions for the insects and fishermen are combined, fishing sites are generally close to each other.

Identifying one or several fishing sites from the road does not guarantee the presence of insects, nor their abundance. The quality of the sites is often different from one place to another. Before buying a licence and shortly before the beginning of the season, the fishermen commonly go to the river to check the presence of insects by lifting a few stones. The fishermen often prioritize the best places from the previous year in the first place. If the river’s morphology hasn’t changed due to construction work, if the stones have not been moved by flooding caused by a typhoon, they are likely to house insects again.

When the fishing season begins, the fishermen go to the sites that were previously identified and, at each one they gather a first catch with the four-hand net. The fishermen often try several locations in the same section of the river because the density of insects can vary from one place to another. The fishermen compare the various sites along the river and a site that has few insects will be abandoned quickly in favour of another more abundant one. Depending on the size of the sites and the initial results from two to five sites can hence be retained by each fisherman, for each season.

The fishermen concentrate their attention on the small rapids, the sections where the current is neither too strong, nor too slow, and in these, they prefer the stones partially covered in green algae. However, from the road, like from the bank, it is hard to assess the composition of the bottom visually. The fishermen then go into the water and walk in the river’s current to assess the stability of the riverbed under their feet. They look for the most stable stones (*shizumi-ishi*), those that are slightly buried in the ground because they know, from experience, that these house the insects. In the same way, while they are fishing, they will avoid places where the stones are the most unstable (*uki-ishi*). These, which often form the strongest rapids, leave large spaces between them which are more favourable for fish (such as sweetfish, *Plecoglossus altivelis*) than insects. They also accumulate a large quantity of debris and detritus (decaying vegetative material mainly) that obstructs the net and complicates the insect sorting.

There are few fishermen, and in season each one finds one or several sites to prospect. If the insects are abundant and the site wide enough, two or even several fishermen may decide to fish the same section of river together. In their search for the best sites, it is not rare either for two fishermen to share the same section of river without it being possible to know which of the two was the first to discover it.

### Moving the fishing in the river, from rapids to rocks

The second stage is the fishing. Since the conditions are tough, once they are in the water, the fishermen want to take the best advantage of their presence and fish quickly. Depending on their size and the abundance of the insects they find there, the sites will be prospected over several days. On each site, a fisherman will define a fishing area for the day and for the following days.

The fishermen organize their movement in the river in different ways, but many choose to advance in the current following the lines that go from downstream to upstream. They begin by the line that is supposed to be the best for the insects, along the small rapids (or riffles). With their equipment, they follow the stones and rocks that make up the bed of the rapids. Although their movement on the line can vary slightly depending on the resistance of the rocks encountered on the way, the fishermen try to maintain a depth of 20 to 30 cm and avoid deviating towards sectors that are too deep where the four-hand net cannot hold the current.

**Fig. 3:**
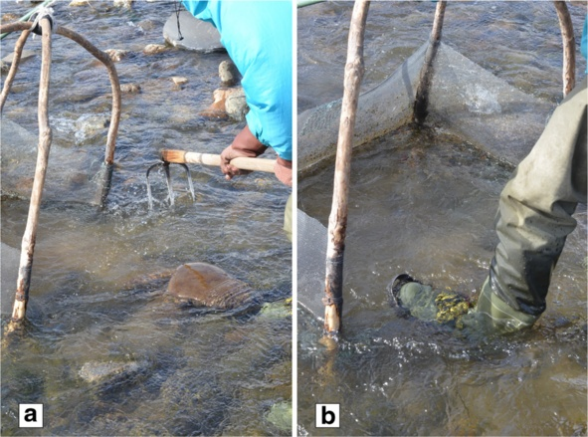
Fishing zazamushi: **a** turning stones upside down, **b** “trampling” the riverbed with metal shoes. (Photos by N. Césard)

The first location is often the furthest one downstream. To place the four-hand net on the bed, the fisherman must first move a few large rocks. For this he uses agricultural tools, generally a hoe (*mannou-kuwa*), sometimes a pick (*tsuruhashi*) or a fork (*sanbon-ba*). Once the space is cleared, the two back branches of the four-hand net are pushed into the riverbed, sometimes with the help of a long mobile foot, the frame and the steel net are inclined forward, the open side of the net is placed on the bed facing the current. Equipped with his tool, the fisherman positions himself upstream from the four-hand net and turns over the stones one by one (Fig. 3).

The area prospected depends on the current, because the drift of the insects also depends on it. If the current is strong, the fisherman can begin between 1 and 2 m away from the four-hand net, to move closer gradually to its opening. If the current is weaker, he begins by turning over the closest stones to the net, from the opening, and progresses by about 1 m upstream. The stones are turned over, generally opposite the net, in a forward to backward movement which lets the water rush under them and the current pushes the insects, debris and detritus towards the four-hand net.

Some of the larvae of interest to the fishermen, the Trichoptera are carried directly towards the net when the large stones are moved, but most of the larvae remain attached under the stones. The fishermen then proceed differently to force the insects to leave their retreat of debris and silk. They observe the size of the stones and the depth: if the stones are broad and visible, the fishermen can either “scrape” (*kaku*) their surface with their tool when they turn them over, or turn them all over and then come back and scrape them with their tool or their heels. The best equipped fishermen then use other equipment under their boots: metal fishing shoes (*kanjiki*) (see Fig. 3).

Once they have been scraped, the stones are pushed to the sides, to a passage that has already been prospected, or to the side that is not prospected, where the current generally runs faster. When the insects have fallen into the water and the path of the largest stones been liberated, the fisherman positions himself in front of the four-hand net, and leaning on it, or on his tool, “treads” (*fumu*) methodically on the riverbed: he moves his feet, which are apart, back and forth, one after the other, in a swinging motion that certain fishermen compare to dancing steps. Some of the stones may not be too large, in which case, the fishermen don’t use their hoe to make the insects fall from the overturned stones, they trample instead on them directly with their metal shoes, using their tool mainly to lean on. By tramping on the riverbed with their metal shoes, the fishermen squash the retreats that have fallen onto the bed and free most of the larvae into the current. For this reason, the submerged part of the steel net, the one open to the current, must cover the riverbed as closely as possible to avoid the insects passing under it.

The riverbed is generally treaded for 2 or even 2 min, over an area that covers, depending on the strength of the current, the opening of the four-hand net and goes 1 or 2 m upstream. The dimensions of the four-hand net and the quality of the net thus play an important part and reflect their use. Certain owners prefer broad, bulkier frames, which offer a wider opening to the current (from 1 to 1.20 m) which allows them to move away from the four-hand net; others use smaller frames (around 1 m) on which they can lean, but which lets them work less far away. The steel net must also be solid and supple to adapt to the riverbed and sometimes stones.

The technique is efficient. The fine steel wire (mesh width 2 mm) allows the Trichoptera larvae being sought to be caught, but also many other organisms and inanimate matter. The analysis of the composition of the samples taken downstream from the four-hand net and inside it shows that few insects escape (Table 3). The samples taken during a catch downstream from the four-hand net show that most of the insects that pass beside or through the wire netting are small Diptera larvae (69.5 % of the number of insects captured, see Table 3). Only a few Trichoptera larvae are not captured (only four larvae in our sample). As observed by the fishermen, catches with the four-hand net are likely to be larger than when the fishermen prospect close to the four-hand net.

**Table 3:** Insects escaping from the four-hand net^a^

Class	Order	Family	Species	No. of individuals (%)	Wet weight (%)
Insects	Ephemeroptera	Potamanthidae		−	−
		Baetidae		0.4	0.05
		Ephemerellidae		12.5	27.2
		Heptageniidae		4.8	6.8
	Plecoptera	Perlidae		0.04	0.5
		Taeniopterygidae		−	−
		Nemouridae		0.1	0
		Capniidae		0.1	0.05
		Others		0.3	0.05
	Hemiptera	Aphelocheiridae		−	−
	Megaloptera	Corydalidae	*Protohermes grandis*	−	−
	Trichoptera	Hydropsychidae		7.1	13.2
		Stenopsychidae	*Stenopsyche marmorata*	0.2^b^	6
		Rhyacophilidae		1.4	1.8
		Others		0.04	0.05
	Coleoptera	Elmidae		0.04	0
		Psephenidae		3.7	11.1
	Diptera			69.5 (Est.)	33 (Est.)
				Total: 2269	Total: 18.7 g
Crustacea	Amphipoda			−	−
Annelida	Oligochaeta			−	−
Platyhelminthes	Tubellaria			−	−
Pisces			*Rhinogobius flumineus*	−	−

^a^The fishing session lasted 9 min in total. The fisherman prospected the riverbed one meter and half away from the four-hand net. Insects were caught behind the four-hand net with a seine net (size: 400 cm (long), 70 cm (high), 1 mm (mesh size). Insects escaped from both sizes on 20 cm

^b^All instars are of stage 5

A fisherman progresses through a few square meters of the river in this way each day and can prospect an area of about 20 square metres in a morning, if the stones are not too large and if the weather conditions are good, or over a full day if the weather is bad and the stones are large. If the site promises more abundant catches, the fisherman will come back the following day, slightly upriver from where he had prospected the previous time. A the end of a rapid, or of a section of river, the fishermen follow parallel lines to either side of the main line of the rapids. Once the rapids of a site have been prospected, the fishermen change site and don’t come back to this one for the rest of the season.

### Sorting the insects, from the grader to the kitchen

When the insects have been caught, the third stage consists of selecting those to keep. Once the place has been trampled on, the fisherman raises his net with the help of the stake which is planted in the riverbed to hold it firmly in place. The collected material is composed of a multitude of insects and what the fishermen called “trash” (*gomi* or *kuzu*). The fisherman then sorts the material a first time. Some of the organic debris, the most visible such as branches, large leaves, but also small fish, are quickly removed by hand and thrown into the river from the four-hand net. This debris contains insects (8.47 % of the total weight of the amount taken in our example), mainly Ephemeroptera (44.83 % of the number of insects caught) and Diptera (40.33 %) (see Table 4).

**Table 4:** Insects and other organisms removed as “trash” on site^a^

				From the four-hand net^b^		From the grader	
Class	Order	Family	Species	No. of individuals (%)	Wet weight (%)	No. of individuals (%)	Wet weight (%)
Insects	Ephemeroptera	Potamanthidae		−	−	0.02	0.01
		Baetidae		0.33	0	0.5	0.01
		Ephemerellidae		29	36.67	35	46.51
		Heptageniidae		15.5	37.63	11.9	16.9
	Plecoptera	Perlidae		0.16	0.6	0.15	0.9
		Taeniopterygidae		−	−	0.02	0
		Nemouridae		−	−	0.02	0
		Capniidae		−	−	−	−
		Others		−	−	0.02	0
	Hemiptera	Aphelocheiridae		−	−	0.04	0.92
	Megaloptera	Corydalidae	*Protohermes grandis*	−	−	0.02	0.02
	Trichoptera	Hydropsychidae		5.33	4.94	5.23	4.65
		Stenopsychidae	*Stenopsyche marmorata*	0.16	3.86	0.19	1.98
		Rhyacophilidae		1	0.24	1.4	1.21
		Others		0.33	0.12	0.21	0.1
	Coleoptera	Elmidae		−	−	0.08	0
		Psephenidae		7.83	10.73	6.97	16.02
	Diptera			40.33	5.18	38.13	11.57
				Total: 600	Total: 8.29 g	Total: 5192	Total: 74.08 g
Crustacea	Amphipoda			−	−	6	0.16
Annelida	Oligochaeta					1	0.01
Platyhelminthes	Tubellaria			1	0.03	9	0.02
Pisces			*Rhinogobius flumineus*	1	0.13	2	0.49
Total debris/detritus weight (wet):					91.25 g		578.57 g (Est.)
Total weight (wet):					99.7 g		723.02 g (Est.)
Debris/detritus weight (%):					91.52		80.02 (Est.)
Insects and other organisms' weight (%):					8.47		19.97 (Est.)

^a^Unwanted or sometimes non-visible organisms removed with inanimate matter (debris, detritus)

^b^We give here the results of one catch (less than 2 min of fishing). The fisherman did four catches in total (before pouring the harvest on the first sieve of the grader)

Then, once the main organic debris has been removed, with a small metal strainer the fisherman collects the catch with several sharp blows. A mixture of insects of various sizes, small debris and detritus, the heap is put into a round basket (*biku*), with its bottom covered with a sheet of plastic, which the fisherman carries hung from his waist. The fisherman then continues his progression along the line of the rapids and repeats the preceding gestures, filling his basket at each stage (less than two minutes between each catch in our example). To help them sort the insects, some fishermen have a second basket inside the first one hung from the waist, into which it fits. It is made of an iron grid. Its purpose is to allow the larvae trapped in the pile of debris to crawl outside the grid and to fall into the main basket.

The main sorting takes places in the grading sieve (*senbetsuki*). After several catches have been transferred from the four-hand net to the basket, the fisherman goes to the grader on the side of the river. He pours the contents of the basket into it and spreads the insects, debris and detritus in the first sieve, again rejecting some undesirable elements. If he uses a grid in his basket, the debris is poured into the grader and the larvae are placed in another container.

**Fig. 4:**
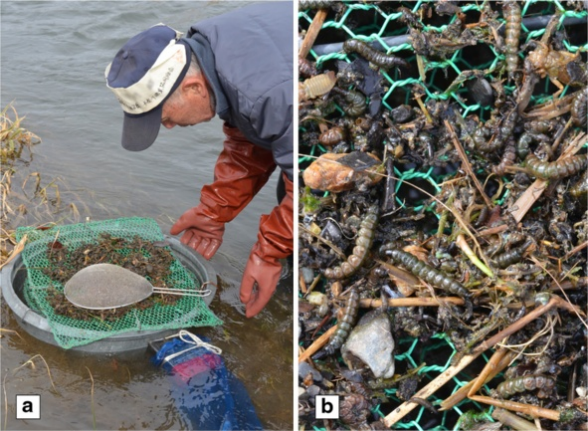
Processing zazamushi: **a** the catch is spread on the first sieve of the grader, **b** the catch is composed of mixture of insects, debris and detritus. (Photos by N. Césard)

Submerged for a third of its height, the grader is a large round container, generally of metal, and open to the current both upstream and downstream (Fig. 4). The main section is composed of three to four sieves with mesh grids that are more and more fine. Depending on the quantity of debris and detritus, the outside temperature the time available to them, some fishermen only use two meshes, others use four.

The first mesh (mesh width 1 cm) holds most of the vegetation debris, the larvae and the less mobile insects (Fig. 4), but also large larvae such as Megaloptera. The second sieve (mesh width 5 mm) retains the small stones above all and some of the Trichoptera larvae that pass through the first sieve. The third sieve (mesh width 3 mm) retains the small mobile larvae that pass through the larger ones, but few Trichoptera. The fishermen rely on the sun to warm up the debris of the first sieve and the insects in it. Since the heat upsets the larvae, they try to flee: either by escaping through the mesh of the sieve, or by crawling out of it, they then fall into the bottom of the grader.

When the weather is cloudy or the fishermen are in a hurry to go home, the larvae are sorted by hand using chopsticks directly from the first sieve, or from a separate container on which the fishermen will have already spread out the insects and debris. Quite often, the larvae taken directly from the sieves contain organic debris and require more sorting after returning home than those that have fallen into the bottom of the grader and swept by the current.

The larvae that manage to pass through the sieves fall into the underwater part of the grader and are carried off in a small net with a tight mesh (mesh width 1 mm) located at its downstream end. The net and its contents constitute the final product of the fishing. The Trichoptera larvae and also several other smaller larvae remain prisoners in the pocket. Some of the inanimate matter that has fallen from the sieves into the bottom of the grader is carried off by the current and disintegrates in the net, but the insects can still hold some. In good seasons, 2 to 3 kilograms of insects can thus be catched during each day of fishing.

Once the various sieves have been emptied of the insects being sought, the fisherman takes them out of the grader one by one and throws the debris and detritus away (into the river or on the ground). This trash still contains several small insects (an estimate of 19.97 % of the total weight of the catch for a fishing session of 9 min, see Table 4). Most are Ephemeroptera (47.42 %) (63.43 % of the biomass of the collected insects) and Diptera (38.13 %) (11.57 % of the biomass of the collected insects, see Table 4).

Back at home, sometimes directly at the boutique, several treatments are still required to separate the insects from the inanimate matter. Depending on the quantities of insects, debris and detritus caught, the conditions of the sites and the river at the time of the fishing, as well as the sorting that has already been done, the preparation of the insects can be shorter or longer and of varying fastidiousness. The washing and initial sorting is generally done by the fisherman and his wife. Some traders prefer however to carry out the various stages themselves.

The catch is first of all boiled, then poured into a strainer and passed through clear tap water to remove the stones, sand, small dead insects and other inanimate matter from it. Boiling allows the larvae to free the small pebbles that they can hold between their legs and claws and to empty some of the contents of their intestines. If most of the debris, detritus and the smallest larvae flow away in the rinsing water, the large undesirable insects are sorted after boiling using chopsticks or gathered on the surface with a small strainer. Many larvae are not retained. In our example these were mainly Ephemeroptera larvae (67.74 % of the biomass of the insects, of which 58.18 % of Ephemerellidae), but also larvae of Coleoptera (Psephenidae, 14.83 % of the insect biomass) and of Trichoptera (Hydropsychidae, 11.12 % of the insect biomass, see Table 5). Boiling then rinsing can be repeated a second time, even a third if the fisherman or the trader considers that the insects still contain detritus.

**Table 5:** Insects removed back home

				First sorting^a^		Second sorting^b^	
Class	Order	Family	Species	No. of individuals (%)	Wet weight (%)	No. of individuals (%)	Wet weight (%)
Insects	Ephemeroptera	Potamanthidae		−	−	−	−
		Baetidae		−	−	−	−
		Ephemerellidae		58.18	62.58	16.7	21.62
		Heptageniidae		3.21	5.16	−	−
	Plecoptera	Perlidae		−	−	−	−
		Taeniopterygidae		−	−	−	−
		Nemouridae		0.29	0.16	−	−
		Capniidae		−	−	−	−
		Others		−	−	−	−
	Hemiptera	Aphelocheiridae		0.29	0.64	−	−
	Megaloptera	Corydalidae	*Protohermes grandis*	−	−	−	−
	Trichoptera	Hydropsychidae		12.57	11.12	58.33	56.7
		Stenopsychidae	*Stenopsyche marmorata*	1.46^c^	4.03	16.7^c^	18.91
		Rhyacophilidae		0.29	0.16	0.29	0.16
		Others		−	−	−	−
	Coleoptera	Elmidae		−	−	−	−
		Psephenidae		19.59	14.83	8.33	2.7
	Diptera			4.08	1.28	−	−
				Total: 342	Total: 6.2 g	Total: 12	Total: 0.37 g
Crustacea	Amphipoda			−	−	−	−
Annelida	Oligochaeta			−	−	−	−
Platyhelminthes	Tubellaria			−	−	−	−
Pisces			*Rhinogobius flumineus*	−	−	−	−

^a^Sorting from the mesh. After first boiling and rinsing

^b^Sorting from the mesh. After second boiling and rinsing

^c^Two instars of stage 5 and three of stage 4

**Fig. 5:**
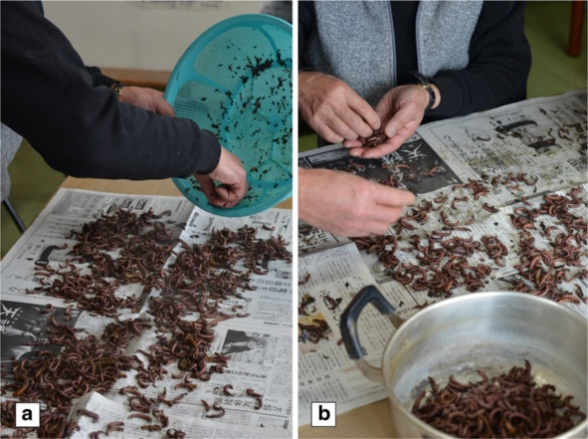
Processing zazamushi: **a** after boiling and cleaning, the insects are spread to be sorted a final time by size and quality, **b** the insects still contain small debris and detritus. (Photos by N. Césard)

Once they have been cleaned, the fisherman or the trader spreads the insects by hand on a working surface, generally on newspaper sheets, then sorts them according to their size and condition (Fig. 5). Like the previous stages, the sorting varies depending on the people, their aims, but also their patience. Some traders sort the larvae by size and species. They then only keep the final stage larvae of Trichoptera and Megaloptera which they separate for sale as two distinct products. Other boutiques don’t want to separate the insects, nor distinguish the stages, and include both species as well as other smaller larvae in their preparation, including Plecoptera (Table 1). Most fishermen and traders do not keep larvae that are too small or damaged. An example of a fisherman sorting for a boutique shows that to obtain a final product of 500 g of both *S. marmorata* and *Protohermes grandis*, 68.34 g of insects and debris were rejected, including 24.8 g of *S. marmorata* larvae (Table 6). Some can decide before cooking to keep a few small insects. In our example (individual consumption), the insects not selected once back home were mainly Trichoptera (Hydropsychidae, 56.7 % of the biomass of the insects), just as certain Ephemeroptera (probably forgotten in the earlier sorting) and a few small larvae of *S. marmorata* (see Table 5).

**Table 6:** Post-catch sorting in weight (wet) (commercial composition)^a^

Type	Species	Sorting after boiling and rinsing	1st sorting (spread on table)	2nd sorting (spread on table)
Insects		2.68	38.07	4.08
	*S. marmorata*	1.71	20.98^b^	2.11^c^
Other debris		1.57	19.24	2.70
Total		4.25 g	57.31 g	6.78 g

^a^Insects and inanimate matter rejected, final product 500 g (including *S. marmorata* and *Protohermes grandis*)

^b^Five final instar damaged individuals

^c^Eighteen individuals (from instar 2 to final instar) too small or damaged

Both traders and fishermen who sell prepared zazamushi pay great attention to the quality of the insects. Most traders prefer to buy the resource fresh to clean and prepare it themselves. Consequently, the majority of the fishermen interviewed who sell their catch do not prepare or cook the insects themselves, and most of them limit themselves to boiling their harvest (mainly to kill the insects and avoid them killing each other or damaging themselves), and then removing the undesirable elements from them (see Table 6). According to the traders, the quality of the insects cooked for consumption depends on their freshness and therefore on the speed with which they are boiled and prepared once they have been fished. Certain boutiques will collect the insects from the fishermen, others ask the fishermen to bring them as quickly as possible. Traders often develop and maintain relationships of trust with the fishermen which guarantee the exclusivity of the harvest and the quality of the insects (and fixed prices for the fishermen).

**Fig. 6:**
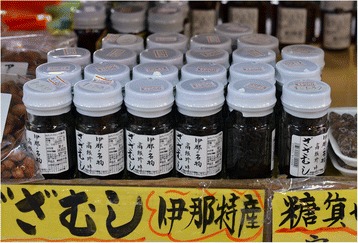
Zazamushi are cooked with sugar and soy sauce and packaged. A glass bottle contains approximately 30 g of insects (Photo by N. Césard)

The insects are cooked and slow-simmered with soya sauce and sugar (in *tsukudani*). Each food souvenir boutique has a different manner of preparing with its own cooking time and specific dosages to retain the flavour (what certain describe as the taste of the Tenryū River). Some prefer to cook small quantities and freeze the boiled and cleaned insects to respond to demand all year round. However, at the beginning of December, the larvae sometimes retain a little sand which is hard to detect before the final cooking. When they find a sandy taste in them, the traders then ask the fishermen to delay their fishing for a few days. In the same way, it is not unusual for them to refuse catches containing a large quantity of debris and detritus and which require long cleaning. For several years, the insects have been bought from the fishermen for JP¥6,000/US$48 per kilogram (more if the insects have already been cleaned) and are resold in boutiques for JP¥1,200/approximately US$10 for a 30 g bottle (Fig. 6).

Our research shows that in the course of the fishing process (Table 4) and then back home (Tables 5 and 7), only a small part of the whole catch is kept (1.57 %, i.e., 98 insects of a total of approximately 6244 insects catched), the wet weight of the insects retained for consumption (28.02 g) representing in our example approximately 3.29 % of the total weight catched (insects, debris and detritus collected at the various stages).

**Table 7:** Final product (individual consumption)^a^

Class	Order	Family	Species	No. of individuals (%)	Wet weight (%)
Insects	Ephemeroptera	Potamanthidae		−	−
		Baetidae		−	−
		Ephemerellidae		−	−
		Heptageniidae		−	−
	Plecoptera	Perlidae		27.55	4.05 (Est.)
		Taeniopterygidae		−	−
		Nemouridae		−	−
		Capniidae		−	−
		Others		−	−
	Hemiptera	Aphelocheiridae		−	−
	Megaloptera	Corydalidae	*Protohermes grandis*	3.06^b^	1.53 (Est.)
	Trichoptera	Hydropsychidae		−	−
		Stenopsychidae	*Stenopsyche marmorata*	69.38	22.44 (Est.)
		Rhyacophilidae		−	−
		Others		−	−
	Coleoptera	Elmidae		−	−
		Psephenidae		−	−
	Diptera			−	−
				Total: 98	Total: 28.02 g
Crustacea	Amphipoda			−	−
Annelida	Oligochaeta			−	−
Platyhelminthes	Tubellaria			−	−
Pisces			*Rhinogobius flumineus*	−	−

^a^Final insects retained, weight before cooking

^b^Two instars quite small and one large

## Discussion

When looking at a mode of harvesting, it is important to determine whether it is “traditional” or a recent innovation [53]. The information gathered from fishermen and the literature suggests that the current technique corresponds to an evolution in subsistence and recreational collecting towards more systematic and organized harvesting of insects whose aim is mainly economic. Fishing in rapids and the use of the four-hand net respond to and accompany the commercial demand for insects. This passage to intensive gathering of insects using more and more specialized equipment relies on a range of knowledge and skills relating to the ecology of the insects and their natural environment which seemed interesting to us to specify in order to understand the recent evolution of the harvest and the composition of the insects sold for consumption.

Adapted for fishing by collectors, themselves more or less regular fishermen, the four-hand net has allowed the number of insects caught to be increased considerably, first on the riverbanks, and then progressively in the small rapids where the insects, Trichoptera larvae amongst others, are more common. In parallel, the morphology of the prospected sites, the abundance of stones in particular has prompted the fishermen to use other tools, such as the hoe and to develop new techniques for gathering and sorting, such as the grading sieve. Indeed, if the use of the four-hand net in riffles has multiplied the quantity of insects caught, it has also had the collateral consequence of increasing the amount of “trash” collected, including many insects other than the target species.

Historical research and interviews with elders show that the four-hand net and its use are inspired and derived from an older piece of fishing equipment, which has now disappeared, used locally to catch small fish and shrimps close to the bank. In the past, fishermen of the region used to capture zazamushi with a broad flat-bottomed bamboo basket (called a *seseri*) which they moved close to the bank or in the shallow branches of the river. Placed in a moderate or weak current, it allowed the larvae downstream of the prospected site to be retained, in particular fragile stoneflies. We suggest here that the use from the 1940s of the metal four-hand net has transformed the harvest that until then had been practiced, by distancing the collectors from the small rivers and the banks of the Tenryū River, first by a few metres, then gradually towards the interior of the river, where the current becomes stronger. In prospecting new sites, the collectors would have thus increased their catch but also changed its composition considerably.

The development of materials used for insect fishing reflects this movement of the collectors towards riffles and the adoption of a more appropriate fishing tool. Over the years, wood and now metal, have replaced bamboo, which is light and fragile. A stainless steel net, which is heavier, allows the bottom to resist the current and the structure serves as a support for the fisherman when he “tramples” the riverbed. The mesh grader was inspired in the same way from a technique used previously, probably for smaller quantities. In the past, the content of the baskets was laid out on a board from which the larvae escaped to fall into a container placed underneath. Still today, fishermen try to make their tools more efficient. Although most have inherited their equipment from a former fisherman, often a relative, the various elements received have been modified and adapted by the new owner. The metal equipment such as the four-hand net, metal shoes and graders, are made by the fisherman or commissioned from craftsmen. Thus, it is common for a fisherman’s idea for improving his own equipment to be taken on by another.

Analysis of the various stages of fishing shows that the choice of insects to be fished begins at the searching stage for fishing sites. The technique of the four-hand net in small rapids allows abundant harvests, but requires thorough sorting of the insects, debris and detritus. In this process, *Stenopsyche marmorata* larvae are systematically retained, as are *Protohermes grandis* larvae encountered during the selection process (see Table 1). Our results show that although the insects are gradually separated at the fishing site from the debris and detritus in which they are mixed, the final selection is made at the time of cleaning, in other words for the fisherman, back at home or in the trader’s kitchen. The composition of insects kept for consumption and sale, traded zazamushi, depends then on an insect selection process which is still broadly individual as is reflected by the different compositions sold by the souvenir boutiques.

In eliminating undesirable debris, but also insects at each stage, the fishermen, like the traders, choose to retain only the insects in which they are interested. Numerous other insects, often smaller ones or those which are too hard to be consumed, are rejected. The selection process being, as we have seen, long and tedious, some traders (see Table 1) can chose to leave in the various stages and in the final selection Plecoptera larvae (mainly *Kamimuria tibialis*), Hydropsychidae (Trichoptera) larvae and the intermediate instars of *S. marmorata*. The example described in detail here reflects in its final stage (Table 7), i.e., when the insects are cleaned a last time before being cooked and kept for self-consumption, the fisherman’s preference. Although our fisherman rejects Hydropsychidae larvae, he selects *S. marmorata* larvae, *Protohermes* larvae and also many Plecoptera larvae, insects that he does not keep when he cleans zazamushi for trade (see Table 7).

The idea that the composition of the commercialized zazamushi would follow the faunal composition is partly contradicted by the scientific literature. Analysis of past and present catches shows that the souvenirs are different based on the people who prepare and sell them, but also that the benthic fauna and zazamushi sold evolve separately in time. Comparison of the various souvenirs sold shows a gradual reduction in the number of species retained (Ephemeroptera and Plecoptera amongst others) while their presence is confirmed in certain sectors of the river in 1934 [34], then again in 1996–97 [26] and in 2007 [25]. The literature also shows that, although as Ueno [27] claims, the Tenryū River had a large population of Plecoptera at the beginning of the century, *Stenopsyche marmorata* has always dominated in compositions sold. This predominance of Trichoptera and *Stenopsyche marmorata* larve in particular, is not recent, but has taken shape over the years and with selection.

Here we see that the main difficulty in fishing for aquatic larvae for human consumption is not so much to gather the insects as to separate those to be retained from the large amount of organic matter in which they live and which they feed. Techniques adapted for collecting, sorting and preparing the insects for their different uses in Japan as elsewhere [10], need to consider the abundance of the various species, the environments the latter occur in and the quantities of them removed from nature. For many people to do this requires a significant investment of time, especially after the harvest, and can constitute a major constraint to consumption, even sale and trade.

## Conclusion

The development of more effective techniques has encouraged longer harvestings and allowed in season a regular supply of insects for the few boutiques that sell them all year around. The resource is abundant and, despite commercialisation and the prices offered, gatherings and over-harvesting are limited by the small number of fishermen (whether or not they have a permit to fish).

The abundance of catches and the success of the methods are explained by the fact that in prospecting in the bed of the river, the collectors have gone to look for the insects in their main ecological habitat. In using the four-hand net in the rapids, catches of caddisflies increase while those of stoneflies, more accustomed to weaker currents, have become rarer. The composition of insects traded partly reflects this evolution. The increase in the number of caddisflies in souvenirs is not necessarily explained by a decrease in other insect populations due to weirs or long-term pollution, but by the moving of collectors towards new fishing sites that are more abundant in larvae, in particular caddisflies, and the use of fishing tools adapted for these sites.

The gradual reduction in the number of stoneflies in the zazamushi sold can be explained by the low number of catches in the species’ habitat and it can also be explained by a deliberate choice by traders to reduce their number in favour of caddisflies. This choice can mainly be explained by two reasons. The first can be connected to the effective availability of stonefly larvae in the environment. Though harder to find and perhaps also less abundant, traders prefer caddisflies. The second can reflect an evolution of local dietary preferences (and more generally of the Japanese people) for animals with a fattier texture and a stronger flavour. Still today, those that consume zazamushi have their preferences. Some consumers, in particular the oldest ones, prefer stoneflies which are considered to be less fatty than caddisflies (and compare their texture to the dorsal section of tuna), while others only consume dobsonflies (which the traders put aside for them) for their taste, but also for their alleged beneficial effects on health [15].

The abundance of one or several species does not explain the harvesting or collecting alone. Knowledge of the environment, skills, but also the technology and trade gains and opportunities constitute key factors. Explanations of the composition of zazamushi sold as food souvenirs thus appear to be ecological as much as they are cultural and commercial. Although Suwa Lake allows the presence of caddisflies (and many other organisms) and the local population already consumed them in the past, it is the traders who have gradually imposed the insects in the preparations by creating souvenirs, and then by changing their content to retain today only the most abundant species.
